# Explaining deep neural networks for knowledge discovery in electrocardiogram analysis

**DOI:** 10.1038/s41598-021-90285-5

**Published:** 2021-05-26

**Authors:** Steven A. Hicks, Jonas L. Isaksen, Vajira Thambawita, Jonas Ghouse, Gustav Ahlberg, Allan Linneberg, Niels Grarup, Inga Strümke, Christina Ellervik, Morten Salling Olesen, Torben Hansen, Claus Graff, Niels-Henrik Holstein-Rathlou, Pål Halvorsen, Mary M. Maleckar, Michael A. Riegler, Jørgen K. Kanters

**Affiliations:** 1 SimulaMet, 0167 Oslo, Norway; 2 Oslo Metropolitan University, 0167 Oslo, Norway; 3 University of Copenhagen, 2200 Copenhagen N, Denmark; 4 Novo Nordisk Foundation Center for Basic Metabolic Research, 2200 Copenhagen N, Denmark; 5 Aalborg University, 9220 Aalborg Ø, Denmark; 6 Simula Research Laboratory, 1364 Fornebu, Norway

**Keywords:** Machine learning, Cardiology

## Abstract

Deep learning-based tools may annotate and interpret medical data more quickly, consistently, and accurately than medical doctors. However, as medical doctors are ultimately responsible for clinical decision-making, any deep learning-based prediction should be accompanied by an explanation that a human can understand. We present an approach called electrocardiogram gradient class activation map (ECGradCAM), which is used to generate attention maps and explain the reasoning behind deep learning-based decision-making in ECG analysis. Attention maps may be used in the clinic to aid diagnosis, discover new medical knowledge, and identify novel features and characteristics of medical tests. In this paper, we showcase how ECGradCAM attention maps can unmask how a novel deep learning model measures both amplitudes and intervals in 12-lead electrocardiograms, and we show an example of how attention maps may be used to develop novel ECG features.

## Introduction

Deep learning methods have the potential to become essential tools for diagnosis and analysis in medicine. Automatic analysis of electrocardiograms (ECGs) is a field with a long history and many different approaches^1–5^, but recent years have shown that deep learning works better than traditional methods^6^. However, this family of machine learning algorithms may also bring much uncertainty and confusion among the medical practitioners they aim to help because of lacking understanding of how these algorithms work. Despite the impressive results in areas like radiology^7^, dermatology^8^, and cardiology^9–11^, deep neural networks are often criticized for being difficult to explain and for providing little to no insight into why they produce a given result (the so-called "black-box phenomenon")^12^. Since doctors are accountable for their diagnoses, a black-box approach is unacceptable^13,14^.

History has shown that doctors in practice prefer simpler, although inferior algorithms to their neural network-based counterparts, primarily because the simple algorithms are more interpretable^15^. Lack of insight has in some cases of machine learning led to obvious mistakes, which has been overlooked because the black-box decision did not allow for interpretation of the neural network predictions^16,17^. A classic example comes from deep learning in radiology (X-ray of the thorax), where the neural networks effectively distinguished between lung cancer and pneumonia simply by predicting the referring department from various labels in the image and not the relevant parts of the X-ray images. When the network is presented with X-rays without similar department labels, the network fails to distinguish between lung cancer and pneumonia^7^. This study is a good example of a mistake rooted in the differences between training and test data distribution. The neural network learned data-specific features that did not generalize to data from outside its domain. This simple but grave mistake could have easily been discovered with an explanation of the predictions where one could easily have observed what the network recognized as the most important feature for its predictions. Hence, it is clear that we need to understand the decisions of the neural network. In this respect, recent developments in explainable artificial intelligence (AI) have shown progress in shedding light on these black-boxes, which seems imperative if deep learning is to be implemented in clinics^18^. Generally, explanations are produced for image data and classification. In this work, we present a method that can obtain explanations for classification and prediction/regression tasks on non-image data. Specifically, we look at ECG where AI has become an emerging topic, where interpretable and explainable results of both classification and prediction will be crucial for clinical implementation and research.

**Figure 1:**
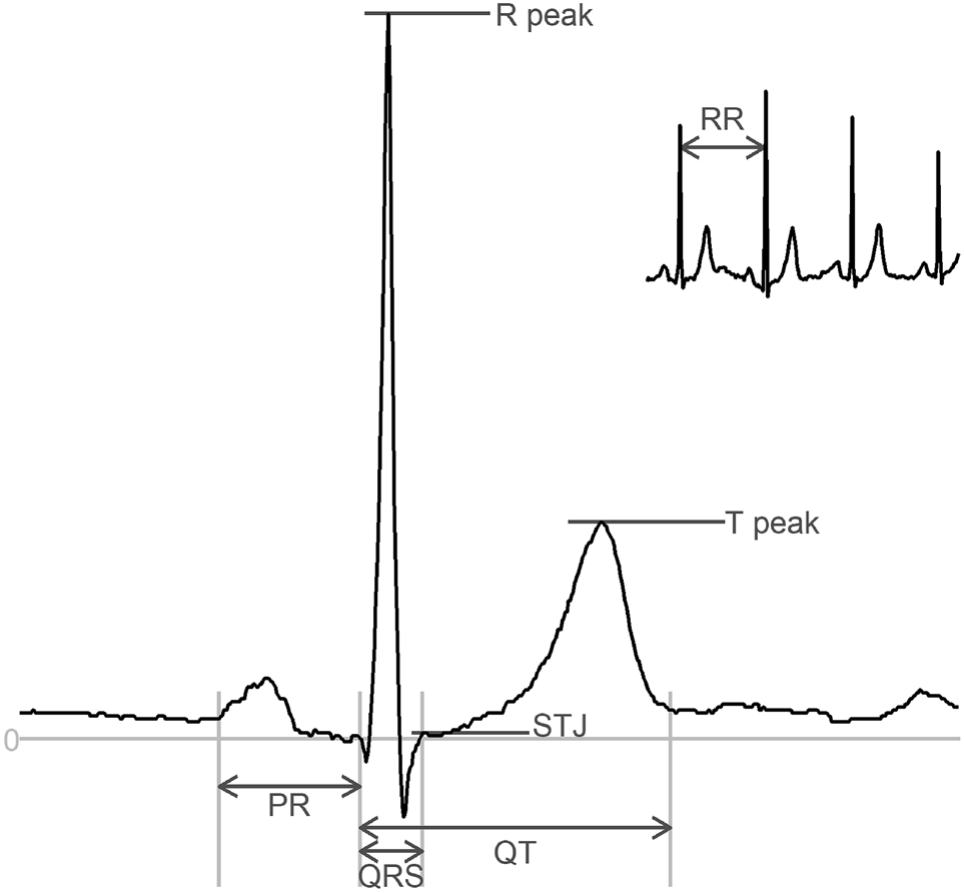
An annotated ECG representative beat and rhythm strip (top-right inset) with intervals (PR, QRS, QT) and amplitudes (R peak, STJ, T peak). Amplitudes are measured with respect to the baseline. STJ denotes J-point elevation. Heart rate is calculated as HR = 60,000/RR where RR is measured in ms.

In the field of electrocardiography, a 12-lead ECG is a recording of the electrical activity of the heart using ten electrodes placed on the patient’s thorax and limbs. The ECG consists of a set of voltage time-series, with several characteristic waves (see Fig. 1), which each carry clinical information about the state of the heart. The timing and the amplitude of these waves contain essential information associated with morbidity and mortality^19–22^. The ECG is one of the cheapest and most commonly used medical procedures, and the availability of large training data sets makes the ECG well-suited for neural network analysis. While automated analysis of ECGs has been a topic of research since the early 1960s^23^, recently, we have seen an introduction of machine learning in ECG analysis. Deep learning has shown to be successful in using features that may indicate cardiac arrhythmias or other diseases^10^.

Incorporating explainability into medical decision-making has three potential advantages. First, for implementing deep learning in the clinic, where medical decisions may be a matter of life and death, a deep learning algorithm that explains how it arrived at a particular decision allows the prevention of rare but potentially fatal mistakes. Such mistakes may be the result of shortcomings in the training of the algorithm (such as biased or narrow training data) or noisy or faulty input data leading to unexpected and extreme decisions. Explainability thus provides a higher level of trust and transparency in the clinical setting because a doctor can understand what the algorithm bases its predictions on^24,25^. This may pave the way for the implementation of neural networks in clinical practice and reduce human error, resulting in fewer fatalities. Second, more explainable models may allow for the identification of novel features that may lead to a new understanding of the disease pathophysiology and increased diagnostic capability, which in the end may save lives. Suppose a deep learning algorithm successfully predicts sudden cardiac death using ECGs from a given population. If the algorithm can explain where the information is located in the ECG, we may combine medical knowledge of the ECG with that location making it possible to identify novel mechanisms of sudden cardiac death. This would potentially make it possible to identify an intervention or possible drug target to prevent untimely death. Third, making the algorithms more interpretable may be important from a legal perspective because one would be able to explain why a model made an incorrect decision and place responsibility accordingly.

The work presented in this paper has three primary contributions. First, we present the architecture of a residual convoluted neural network (CNN) and show that it is able to quantify intervals and amplitudes in the ECG more accurately than trained cardiologists are. Second, we present a modified version of the GradCAM^26^ algorithm called electrocardiogram gradient class activation map (ECGradCAM) and show how the resulting attention maps can be utilized for ECG analysis to understand, interpret, and learn from neural network predictions. Third, we show how network and attention maps may be combined to identify novel features in the ECG by identifying a novel feature to determine the sex of a person based on an ECG.

## Results

### Automatic ECG analysis and data description

We define two case studies for model evaluation: a regression study, measuring standard, clinically relevant intervals and amplitudes from the ECG, and a classification study to predict the sex from the ECG. Numerous cardiovascular diseases are diagnosed by measuring key intervals and amplitudes present in the ECG^27,28^, and we leverage this to predict these intervals directly instead of categorizing ECGs into normal and abnormal groups. The predicted intervals and amplitudes include the PR interval, QRS duration, heart rate, J-point elevation, QT interval, R-wave amplitude, and T-wave amplitude (see Fig. 1). By predicting these measurements directly, we allow for better interpretation of the results rather than limiting it to a predefined set of categories. The second case study looks at differentiating between male and female ECGs. This use case is motivated by the difficulty for humans to determine sex based on ECGs alone, making it a good candidate for visualization as one may find certain features that correlate to sex that have previously gone unfound.

All models are trained and evaluated on either raw 10-s 12-lead ECGs or on the 12SL-generated median beat from the GESUS dataset^29^. The performance of all GESUS generated models is replicated in the Inter99 dataset^30^. The demographics of the study populations are summarized in Table 1. To evaluate the effect of ECG abnormalities on the prediction performance of our network, we tested the prediction errors on subgroups with bundle branch blocks (QRS ≥ 120 ms) or first-degree AV block (PR > 220 ms). Furthermore, to study the performance on ECG with strange, abnormal T waves, we used the existing T-wave Morphology Combination Score^31^ to divide the ECGs into four different groups ranging from peaked to flattened T-waves. Supplementary Table S1 shows that the network performed only slightly worse in subjects with bundle branch block, AV block, and flattened T waves, respectively.

**Table 1:** Characteristics of the participants in both population studies.

Variable	GESUS (training)	Inter99 (replication)
Number of samples (*n*)	8939	6667
Age (years)	56.6 [35.1; 78.4]	45.3 [34.5; 60.0]
Female sex	54.3% (4852)	51.2% (3412)
BMI (kg/m^2^)	26.1 [20.4; 35.3]	25.7 [20.2; 34.8]
Heart rate (bpm)	64 [48; 85]	66 [51; 86]
QT interval (ms)	408 [364; 460]	402 [362; 450]
PR interval (ms)	158 [126; 204]	156 [124; 196]
QRS duration (ms)	92 [76; 118]	90 [76; 110]
J-point elevation V5 (µV)	− 5 [− 54; 48]	4 [− 35; 58]
R-peak amplitude V5 (µV)	1376 [698; 2,426]	1171 [600; 2,044]
T-wave amplitude V5 (µV)	346 [122; 698]	327 [122; 649]
Bundle branch block (QRS ≥ 120 ms)	4.4% (390)	1.6% (107)
1° AV block (PR > 220 ms)	2.0% (174)	0.9% (61)

Values are presented as median [fifth to ninety-fifth percentiles] for continuous measures and % (*n*) for categorical variables.

### CNN results

The performance of our method for predicting ECG intervals and amplitudes is evaluated using quantitative regression metrics, as seen in Table 2. The primary metrics used for evaluation are the mean absolute error (MAE) as it is easily interpretable, and the root-mean-squared error (RMSE) as it is more sensitive to outliers. In Table 2, we see that every model beats the ZeroR-estimate (predicting the mean) by a large margin. This shows that the proposed architecture successfully analyzes the ECG, both in the voltage and time domains. For interval measurements, the MAEs are close to two samples (4 ms) for both the median beat and rhythm strip (10 s) measurements. Amplitude measurements varied similarly (the least significant bit is 4.88 µV), indicating that the network performed equally well with voltage and time-domain measurements.

**Table 2:** Training and validation error in GESUS^25^ and replication error in Inter99^2^.

Type	Variables	Validation on GESUS (fivefold)		Replication on Inter99		Zero R on Inter99	
		MAE	RMSE	MAE	RMSE	MAE	RMSE
Median	QT Interval (ms)	3.26 ± 0.80	5.08 ± 0.40	3.13 ± 0.19	4.89 ± 0.19	21.7	27.4
	PR Interval (ms)	2.82 ± 0.15	4.52 ± 0.49	2.73 ± 0.06	4.70 ± 0.23	17.6	22.8
	QRS duration (ms)	2.98 ± 0.15	4.10 ± 0.22	2.58 ± 0.07	3.43 ± 0.07	9.0	11.6
	Heart Rate (beats per min)	1.54 ± 0.07	2.44 ± 0.09	1.57 ± 0.06	2.33 ± 0.07	8.6	11.1
	J-point elevation (µV)	8.16 ± 0.40	11.20 ± 0.69	5.77 ± 0.10	8.09 ± 0.12	22.2	29.0
	T-wave amplitude (µV)	5.63 ± 1.31	15.2 ± 6.83	5.80 ± 1.13	16.10 ± 0.53	129.0	167.0
	R-peak amplitude (µV)	8.60 ± 1.05	16.00 ± 4.13	8.30 ± 0.98	21.70 ± 0.71	413.0	501.0
Rhythm	QT Interval (ms)	3.97 ± 0.03	6.05 ± 0.39	3.62 ± 0.03	5.82 ± 0.20	21.7	27.4
	PR Interval (ms)	3.67 ± 0.21	5.60 ± 0.60	3.58 ± 0.60	5.80 ± 0.31	17.6	22.8
	QRS duration (ms)	3.08 ± 0.12	4.33 ± 0.17	3.39 ± 0.06	4.49 ± 0.07	9.0	11.6
	Heart Rate (beats per min)	0.31 ± 0.01	0.40 ± 0.02	0.18 ± 0.01	0.6 ± 0.1	8.6	11.1
	J-point elevation (µV)	10.50 ± 0.31	14.10 ± 0.50	7.90 ± 0.19	10.70 ± 0.24	22.2	29.0
	T-wave amplitude (µV)	11.50 ± 0.43	25.00 ± 5.55	9.40 ± 0.41	19.40 ± 1.79	129.0	167.0
	R-peak amplitude (µV)	20.10 ± 0.70	33.00 ± 4.54	17.4 ± 0.55	51.00 ± 13.21	413.0	501.0

### Attention maps

**Figure 2:**
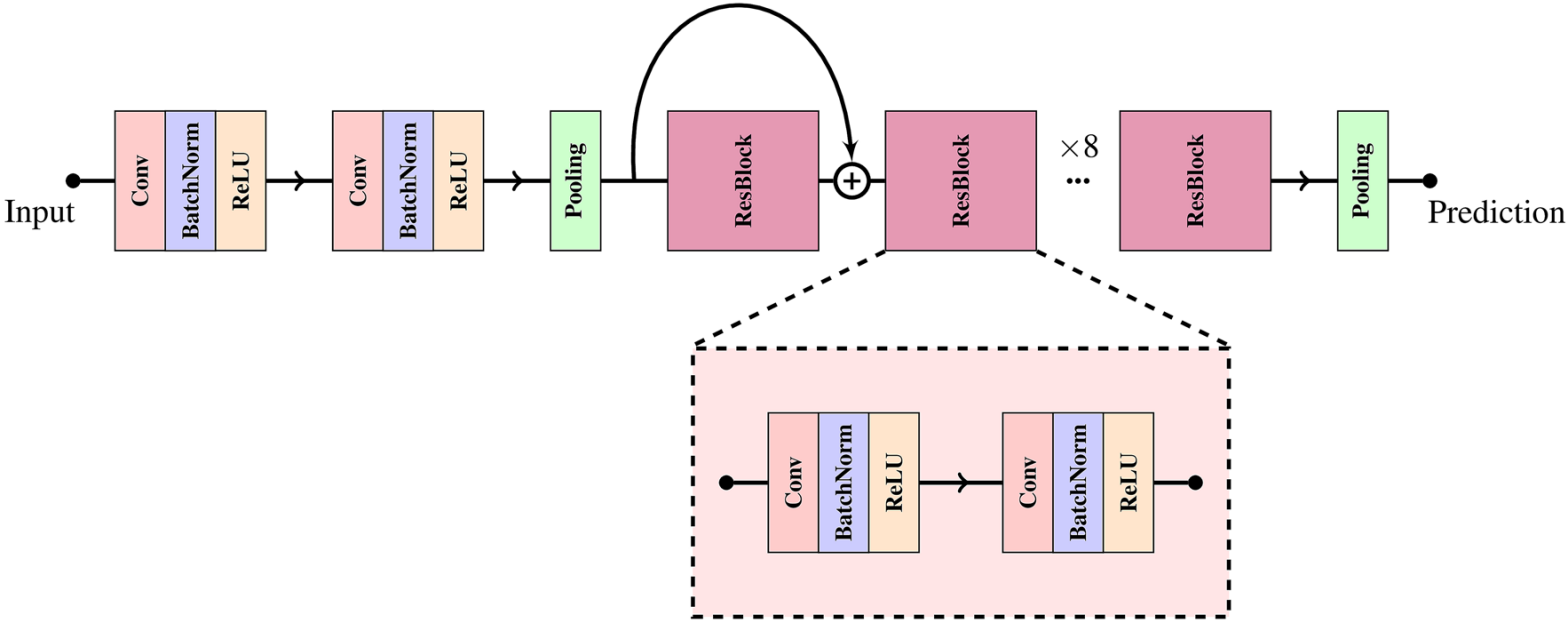
The convolutional neural network-based architecture used for all experiments. Each red block corresponds to a convolutional layer, the blue blocks represent a batch normalization layer, the yellow blocks are ReLU activations, and the green block represents average pooling. Besides these base building blocks, the model consists of eight residual modules (purple blocks) composed of two sequential blocks of convolution, batch normalization, and ReLU activation.

To create meaningful and detailed visualizations, we modified the GradCAM approach so that visualizations are generated for each lead of the ECG, where the final attention maps are produced by averaging the importance values across all leads. We call this method ECGradCAM since it can give a more accurate representation of what regions of the ECG are most important for the model. We focus our interpretation on the last layer of the last residual module of the neural network (as shown in Fig. 2). This corresponds to the final layer before prediction, meaning the visualizations show what areas of the ECG are deemed most important at the moment of prediction. It can also be useful to interpret the intermediate layers of a network^32^ as these layers may offer insight into how the network’s perception changes and how it narrows down the analysis to the final result (see Supplementary Figure S1). Here, we note that the initial residual block recognizes several features in the ECG, which becomes more and more focused on the relevant wave as the ECG progresses through the residual layers.

**Figure 3:**
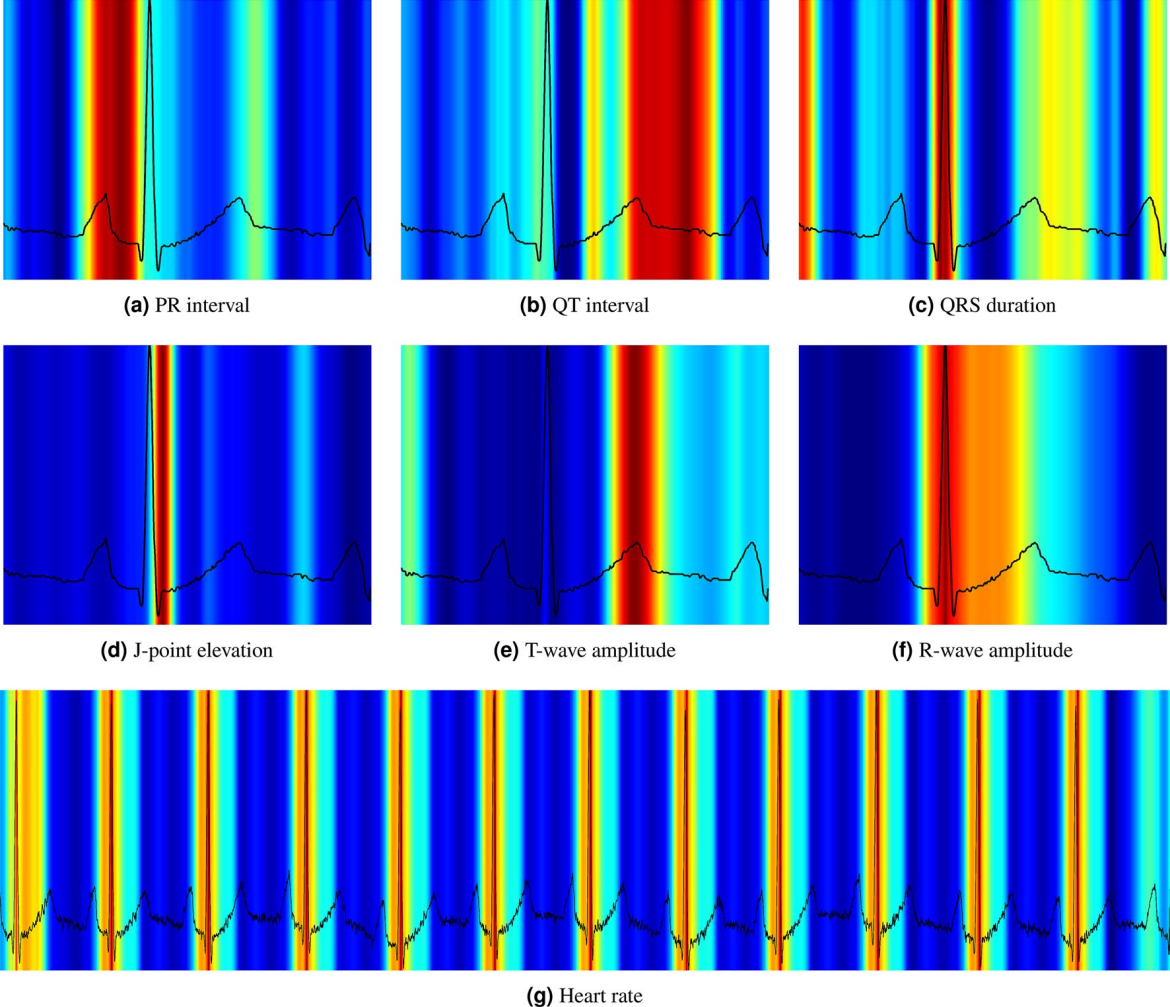
Visualization of the attention maps generated for the interval and amplitude prediction models. As we can see from the plots, the model learns to inspect the waves and intervals that are related to the predicted variable. Red color indicates high importance and blue color low importance of the ECG for the decision of the neural network.

The attention maps often highlight the areas we expect when predicting a specific interval or amplitude. Figure 3 presents a median ECG visualized for six of the predicted variables. For instance, the QRS complex is highlighted when we predict QRS duration, and the end of the T-wave is delineated along with the beginning of the QRS complex for QT interval measurement. For amplitude measurements, the corresponding wave top is correctly pinpointed by the attention maps. One should note that for amplitude measurements, other parts of the ECG are given minor importance, likely for the network to learn about the ECG voltage baseline. For intervals, secondary activations are also observed, such as the T-wave being highlighted when measuring the PR-interval. We hypothesize that these secondary activations may be happening because the network needs to appreciate the whole ECG in order to narrow its search down and perform the actual measurements. This is further supported by the PR interval attention maps generated for the intermediate layers (see Supplementary Figure S1), where the network highlights the QT in the former layers but less so at the moment of prediction.

### Sex prediction

**Figure 4:**
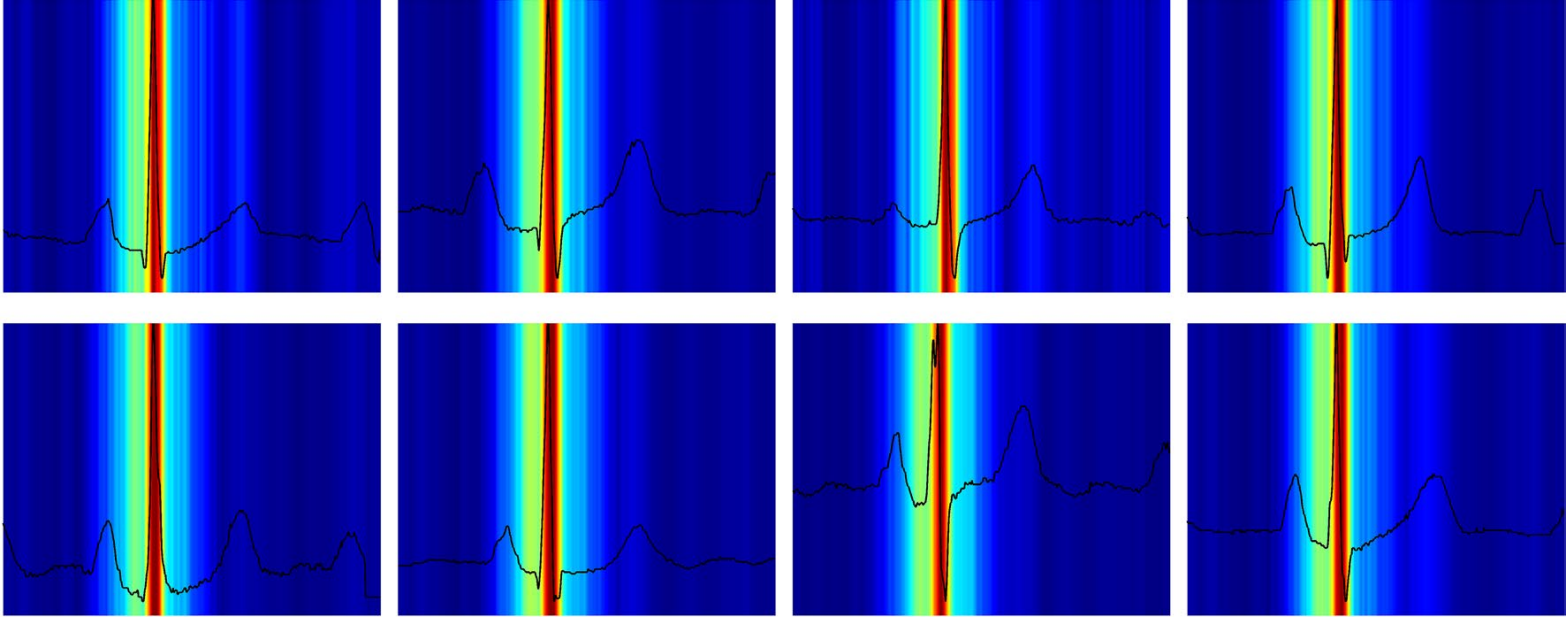
Visualizations of the attention maps from the sex classification model for eight different ECGs. The plots suggest that the QRS-complex and especially the downslope of the R-wave is most important when distinguishing between a male and female ECG. Red color indicates high importance and blue color low importance of the ECG for the decision of the neural network.

For a cardiologist, the task of determining a subject’s sex from the ECG is nearly impossible. However, our network is able to correctly identify the sex with an accuracy of 89% (Table 3). Here, we can see the potential of attention maps, as the accuracy output from the network does not give any clue or insight into how the network made its decision on sex classification. The attention maps (see Fig. 4) clearly show that the ECG sex classification is mainly based on the QRS complex and more specifically on the downslope of the R-wave, offering new insight into electrophysiology. Using findings from the attention maps, we did a post-hoc analysis with logistic regression predicting sex using QRS duration, R- and S-amplitudes, and the timing of the R- and S-waves. It revealed an accuracy of 73% (our CNN: 89% QRS duration alone: 69%) and an AUC of 0.80 (our CNN 0.96 QRS duration alone: 0.72). The wave blocking experiments (Table 3) verified this observation, since removing the P-wave has almost no influence on the accuracy of the sex prediction, removing the T-wave had only minor influence, whereas removing the QRS complex resulted in a drastic reduction in performance. This shows that one can obtain new knowledge by using our ECGradCam method combined with the deep neural network.

**Table 3:** Mean absolute error (MAE) and accuracy (ACC) ± standard deviation measured on the replication dataset when blanking specific waves of a median heartbeat.

Variable	Metric	Median ECG	Blanking P-wave	Blanking QRS complex	Blanking T-wave
QT interval (ms)	MAE ↓	3*.*13 ± 0*.*21	3*.*20 ± 0*.*22	31*.*76 ± 6*.*34	47*.*35 ± 12*.*38
PR interval (ms)	MAE ↓	2*.*73 ± 0*.*06	32*.*95 ± 10*.*31	40*.*55 ± 5*.*88	3*.*84 ± 0*.*71
QRS duration (ms)	MAE ↓	2*.*58 ± 0*.*08	3*.*99 ± 0*.*48	40*.*55 ± 5*.*88	3*.*50 ± 0*.*10
Heart Rate (bpm)	MAE ↓	1*.*57 ± 0*.*07	2*.*92 ± 0*.*19	3*.*62 ± 1*.*89	4*.*79 ± 1*.*10
J-point elevation (µV)	MAE ↓	5*.*77 ± 0*.*18	6*.*43 ± 0*.*56	23*.*07 ± 3*.*29	8*.*62 ± 0*.*42
T-wave amplitude (µV)	MAE ↓	5*.*80 ± 1*.*29	6*.*13 ± 1*.*45	8*.*70 ± 1*.*49	339*.*04 ± 8*.*0
R-wave amplitude (µV)	MAE ↓	8*.*35 ± 1*.*12	8*.*64 ± 1*.*11	927*.*38 ± 16*.*0	10*.*48 ± 3*.*56
Sex classification (%)	ACC ↑	88*.*80 ± 0*.*7	87*.*50 ± 1*.*0	62*.*40 ± 6*.*6	79*.*80 ± 2*.*5

Prediction errors increased dramatically when the feature in question is blanked out. Prediction errors also often increased slightly when other parts of the ECG are blanked. In the metric column, the arrow signifies whether higher or lower values are better, i.e., an arrow pointing downwards means that lower values are preferable.

### Human cardiologist vs neural network evaluation

To assess how the neural network compares to standard clinical decision making, we further evaluate the performance of our model by comparing its predictions to predictions made by cardiologists who have manually annotated a set of twenty randomly selected ECGs from the Inter99 replication dataset. As seen in Table 4, the trained networks prove substantially more precise and consistent than human expert assessments. Human bias-corrected MAE and RMSE are around 15–20 ms, i.e., a factor 4 to 5× higher than the neural network. Errors in heart rate measurements are below one beat per minute (BPM) for the network but about 3 BPM for the human operators with multiple errors above 10 BPM. Amplitude measurements are much more difficult for humans, given the resolution of the digital ECG and the accuracy of the human eye. For the R-peak amplitude, the network operates at an MAE of twice the least significant bit at 4.8 µV. In contrast, the human reviewer operates with an MAE in the magnitude of half a millimeter (corresponding to 50 µV or 10 the times least significant bit).

**Table 4:** Evaluation of twenty randomly selected ECGs by two experienced cardiologists.

Variables	Test set, mean ± SD	Neural network			Doctor A			Doctor B		
		Bias	MAE*	R	Bias	MAE*	R	Bias	MAE*	R
Heart rate (bpm)	71 ± 8	0.04	0.20	0.99	0.98	3.20	0.68	− 0.52	1,38	0.99
QT interval (ms)	392 ± 21	0.50	3.70	0.98	− 8.50	15.8	0.49	− 17.8	9.2	0.89
QRS duration (ms)	91 ± 10	− 3.30	3.00	0.98	− 7.80	11.9	0.39	− 7.6	8.22	0.55
PR interval (ms)	161 ± 16	− 2.50	4.70	0.99	5.90	8.00	0.87	6.45	9.01	0.82
R-peak amplitude (mm)	12 ± 4	0.02	0.16	0.99	0.57	0.42	0.98	0.06	0.50	0.95
J-point elevation (mm)	− 0.04 ± 0.28	0.02	0.09	0.98	0.12	0.19	− 0.01	− 0.01	0.17	0.86
T-wave amplitude (mm)	3.00 ± 1.50	0.03	0.10	0.99	− 0.13	0.32	0.89	0.20	0.56	0.80

Bias is the average difference between the ground truth and the doctor/network. Mean absolute error (MAE*) is the bias-subtracted mean absolute error, to account for the fact that there is no universal correct measurement for an ECG, ensuring that any personal bias does not contribute to the error (e.g., some doctors measure QT consistently shorter or longer than others). R: Pearson’s correlation coefficient between the doctor’s two measurements/the neural networks five folds.

*SD* Standard deviation.

## Discussion

This paper identified three novel findings. First, we presented a residual CNN that reliably analyzes both ECG intervals (time dimension) and amplitudes (voltage dimension) independent of whether the ECG presented is a 10-s 12-lead ECG or a 1.2-s median representative beat. The architecture proved accurate for a variety of different ECG tasks. In all cases, CNN prediction outperformed the cardiologists by a large margin. Furthermore, with repeated blinded measurements, the cardiologists had a large intra-observer variation, whereas the neural network was very consistent in its predictions between folds. The MAE was between 3 and 4 ms corresponding to two samples which is close to the physical obtainable lower limit (since the interval uncertainty consists of two cumulative uncertainties of 2 ms at each the end of the interval). In general, measurements were more accurate when using 1.2-s median beats compared to 10-s rhythm strips, except for heart rate. The generation of the median beat reduces noise by averaging all beats during the 10-s ECG, stretching each complex to minimize the influence of variations, making measurements more accurate. The exception of heart rate is not surprising since several ECG complexes are needed to properly estimate heart rate, and these are only found in the rhythm strip. More surprising is the finding that the neural network uses other features than the RR-interval to calculate heart rate and obtains a relatively good estimate from the single ECG complex of the median beat. In fact, the heart rate estimate from the median was only slightly worse than that based on the rhythm strip (see Table 2). Similar to human overreaders, the network performed slightly worse in subjects with conduction blocks. However, the increase in prediction error was small compared to the human overreader error, showing a satisfactory accuracy for clinical use. The QT interval is an important feature in the ECG because the interval is related to sudden death^33^. It is well known that abnormal T waves are difficult to measure correctly, which is important because these abnormal T waves are associated with increased mortality^34^. As seen in the supplementary (Supplementary Table S1), the network accurately measured even the most flattened T waves only marginally worse than the “easy-to-measure” peaked T waves with a sharp, well-defined end.

Second, we presented ECGradCAM attention maps for 12-lead ECG-analysis to explain how the network made its decisions. In medical practice, explainability is crucial because medical doctors are concerned that algorithms may produce erroneous results, either due to bias or trying to predict outcomes not appropriately represented in the training data. For example, measurement of the PR interval in a case of ventricular tachycardia would be unreliable if the training set only consisted of ECGs taken in sinus rhythm. Interpretation and transparency should be at the forefront when developing new algorithms intended for medical use. Although the results suggest that deep learning could be an essential tool for cardiologists for doing analysis and interpretation of the ECG, it is doubtful that the neural network models without explanations of an estimate of uncertainty would be accepted by doctors.

The attention maps showed nicely that all amplitude measurements focused on the proper ECG wave, and in cases of interval measurements, both the beginning and the end of the specific interval are most often highlighted by the algorithm. The obscurity tests confirmed the attention map results. When we removed ECG waves used for the specific interval or amplitude measurement, the MAE increased dramatically, confirming the message from the attention maps that our CNN focused on the same features as human cardiologists, just more accurately and reliably. One may also notice that the network tries to extract information about the baseline from the ECG. Since we use batch normalization (a standard feature in neural networks to avoid exploding gradients), the network had to get an idea of the magnitude of normalization to restore the absolute values needed for amplitude prediction. This may be why the network also focuses on more steady, constant parts of the ECG. By providing these explanations with a predicted variable, we allow the users to interpret the results with confidence that model had some notion of the traits that make up the variable in question.

Third, sex prediction is an excellent example of how neural networks combined with an explanation method can be used to discover novel medical knowledge. It is well known that there are sex differences in the ECG. Female ECGs, on average, have a longer QT interval, faster heart rate, and shorter QRS duration^35^. However, if one were to ask a cardiologist to determine the sex based on an ECG alone, they would not be able to make a confident prediction. Recent studies using deep learning have shown that neural networks can differentiate between the sexes from the ECG alone^36^, but the underlying reasoning is not provided. The attention maps indicate that the physiological background seems to be differences in the R-wave downslope, which may provide important mechanistic insight into the observed sex differences. We confirmed the findings by our neural network that simple logistic regression with QRS duration, wave amplitudes, and timing (slopes can be inferred by wave amplitudes and timings) significantly improved sex prediction compared to QRS duration alone. Although adding the R-wave downslope to the QRS duration significantly increased sex prediction, the neural network still performed better than the logistic regression, most likely because the R-wave downslope is not the sole source of information. However, it is also possible that the R-wave downslope contains nonlinear information or that only part of the downslope is relevant. This study constitutes a scholarly example that the use of attention maps can assist scientists in discovering novel insights and identify hitherto unknown features for classification, which may lead to important physiological understanding. Classifying an appropriate outcome in a suitable population, one may identify novel prognostic markers in the ECG for that outcome, which may lead to a suggestion for possible treatments.

## Conclusions

This paper presented a study on interpreting deep learning models used for ECG analysis. We propose a neural network architecture that predicts multiple attributes of a standard median or 10-s rhythm ECG with low error. The model was compared against real-world cardiologists, and our model outperformed the cardiologists by a large margin. The predictions were interpreted using attention maps (ECGradCAM), which show how the network operates and confirmed that the neural network analyzes ECGs similarly to trained cardiologists. Furthermore, we show that the neural network can differentiate between male and female ECGs with over 90% accuracy. Using the ECGradCAM attention maps, we find that the down-slope of the R wave is a crucial feature of an ECG when determining sex. This emphasizes the need for more interpretable machine learning methods as they can be used to find new insights in rather mature medical fields such as ECG analysis. We believe that open and transparent systems are paramount for their adoption and use in medicine. Making high-risk decisions based on the output of a black-box algorithm is irresponsible and could potentially have fatal consequences that could easily be avoided. We hope this paper motivates a more thorough evaluation and interpretation of deep learning-based models applied to all of medicine and not only to ECGs.

## Methods

### Data populations

We use digital ECGs from two population studies. (1) The Danish General Suburban Population Study (GESUS)^29^ consisting of 8939 free-living subjects (age 56*.*5 ± 13*.*5, 54% females) at least 18 years old from the Naestved municipality, 90 km south of Copenhagen, the Capital of Denmark, randomly chosen. The study was approved by the local ethics committee (SJ-113, SJ-114, SJ-147, SJ-278). (2) The Inter99 study (CT00289237, ClinicalTrials.gov) consists of 6,667 free-living subjects (age 46*.*1 ± 7*.*9, 51% females) randomly drawn from the Glostrup municipality with an age of 30–65 years^30^. This yields a collection of ECGs from people with and without cardiac disease and an equal representation of men and women. Both studies are conducted in accordance with the Declaration of Helsinki.

### Electrocardiography

All ECGs are digitally recorded as 10-s ECGs with 12 leads. All ECGs are transferred to a MUSE Cardiology Information system (GE Healthcare, Wauwatosa, WI, USA) and ground truths are calculated with version 21 of the Marquette 12 SL algorithm (GE Healthcare, Wauwatosa, WI, USA). The ECGs are recorded with a sample rate of 500 Hz and a resolution of 4.88 µV per least significant bit.

### Prediction model

#### Architecture

A digital electronic ECG can be represented as a two-dimensional matrix of integers representing the voltage at a specific point in time. To analyze these measurements, we use a standard convolutional neural network (CNN) consisting of eight residual modules (as introduced by He et al.^37^) to capture the complex features and relationships present in a standard ECG. The neural network architecture consists of 1,652,993 parameters and is built to handle two different types of input, a single representative median heartbeat of 1.2-s duration and a 10-s rhythm ECG. Both input types contain data from 12-lead ECGs. A detailed view of the neural network architecture can be seen in Fig. 2. From the input layer, the ECG is passed through two convolutional layers before being average pooled. The two convolutional layers generate 64 and 32 feature maps with a kernel size of 8 and 3. After this initial convolution block, the output is sent through eight residual blocks, each consisting of two convolutions. Each convolutional layer in the residual blocks generates 64 and 32 feature maps, respectively, and both layers use a kernel size of 50. We use a large kernel size to extend the receptive field to include multiple parts of a typical ECG. This could, for example, capture both the P wave and the QRS complex in a single convolution. We add batch normalization after each convolution and dropout after the final convolution with a drop rate of 50%. After the eight residual blocks, the output is globally average pooled before making the final prediction. The prediction layer consists of a single neuron with a linear activation that predicts a single variable of the ECG.

#### Training

All models are trained for a maximum of 1000 epochs on a computer consisting of two Intel Xeon Silver 4116 CPUs running at 2.1 GHz, four Nvidia RTX 2080Ti graphics cards, and 96 gigabytes of RAM. The models are implemented using Keras version 2.1.0 with a TensorFlow backend on Ubuntu 18.04.2. Processing one ECG takes about 0.06 s using the aforementioned hardware and Python libraries. To optimize the weights, we used the gradient descent-based optimizer Nadam^38^ with a learning rate of 0*.*0005. The learning rate is selected based on manual testing and prior experience from our previous works^32^. Otherwise, we used the Keras defaults for all optimizer parameters. In total, we performed 14 different experiments, seven using the median for prediction and seven using the rhythm. The variables predicted with regression parameters (include the QT interval, PR interval, QRS duration, heart rate, ST-segment deviation from baseline at the junction (STJ), T-wave amplitude, and R-peak amplitude). The three amplitudes are lead specific and lead V5 is used. Some of these variables cater more to rhythm analyses (such as heart rate), while others are more appropriate for median complexes (such as the R-peak). One problem with training on the median complexes is that they are all centered in a manufacturer-specific way, whereby each wave appears in nearly the same place in each of the ECG. Thereby, the network can learn to predict a particular vicinity and guarantee a relatively low error. To circumvent this problem, we time-shifted all median complexes by a random amount (− 40 to +40 ms) so that the network learns to find the individual waves. This increases the likelihood that the model can be used on ECGs from other manufactures with different temporal alignment. No alignment is performed for the rhythm ECGs; the start of the recording is random with respect to the ECG. Furthermore, to test the network’s ability to classify in binary outcomes, we classified the ECG for sex (male/female).

#### Attention maps

To obtain physiological insights from the neural network’s decisions, it is necessary to understand how and why a decision is achieved. In this study, we used attention maps to visualize which parts of the ECG have importance for each interval/amplitude prediction. To explain the predictions of our model, we use gradient-based activation maps (attention maps) to visualize which parts of the ECG are the most important when predicting a given variable or class. The technique used is a modified version of GradCAM^26^, commonly used to interpret image classification models. As we show in our study, this approach works just as well for regression tasks of quantitative measurements in the data. Visualizations are generated based on a given network layer and output neuron, which produces a heat map that marks the most important areas as hot (red color) and less important regions as cold (blue color). In this context, importance signifies how much weight a specific area contributes to the overall prediction. We are not the first to use attention maps to interpret deep neural networks applied to ECGs^39,40^. Most other works use these visualizations to confirm that their model does not deviate from the expectations of the medical doctors^41^. Our work goes one step further and expands the method of explanations to find new insights into the unique properties of ECGs through a case study on sex classification. Furthermore, even though the attention maps are generated on a per-lead basis, we average the explanations for each lead to produce a visualization that contains more fine-grained details and thus is able to more accurately represent what regions of the ECG are most important for the model when making a specific prediction.

#### Evaluation

To ensure a fair and robust evaluation, we trained each model with five-fold cross-validation for 1000 epochs on the GESUS dataset^29^, resulting in 7152 samples being used for training and 1787 for validation. After training and internal cross-validation, the results of GESUS models are replicated in the Inter99 dataset^30^ to examine whether the models are generalizable or not. As seen in Table 1, GESUS and Inter99 datasets are comparable regarding ECG measurements, although participants in the GESUS study are on average older than participants in the Inter99 study. The neural network performance is evaluated by the MAEs (|predicted – actual value|) and the RMSEs ($\sqrt {\frac{1}{n}\sum \left( {\left| {{\text{predicted }}{-}{\text{ actual value}}} \right|} \right)^{2} }$) to evaluate the mistakes of the neural networks relative to the ground truth. To give an idea of the magnitude of uncertainty, we calculated the ZeroR-estimate, defined as constantly guessing the population mean of the desired variable. If a model’s performance is not better than ZeroR, the model has not learned anything except the population mean. Conversely, if the model performance is better than the ZeroR, it follows that the model has succeeded in extracting and processing features from the ECGs.

Furthermore, ECGs were evaluated manually by two skilled cardiologists. Whereas the neural network by definition has a bias (i.e., average error) of zero (ignoring an eventual bias in the ground truth from the 12SL algorithm), the human overreaders may exhibit substantial bias relative to the ground truth (i.e., the measure consistently shorter or longer intervals), which originates from their own training and personal preference. Since this bias is not an error, the human bias is subtracted from the errors before calculating human MAE and RMSE.

#### Wave blocking

To verify that the neural network model is focusing on relevant waves of the ECG, and as an alternative to the attention maps, we remove specific parts of the ECG (either the P- QRS- or T-wave) from the median ECGs of the replication set. Using the MUSE 12SL fiducial points, we blank out a wave by replacing it from the start to the end with a lead-specific linear interpolation. This analysis represents an alternative measure of explainability for representative beats of an ECG by analyzing the decrease in performance when different waves of the ECG are blanked. Thus, we can test how dependent our model is on different parts of the ECG and verify which waves the model is focusing on when making a prediction. The results in Table 3 show that the model performance drop when the wave involved with a particular feature is removed. However, we also find that removing non-involved waves typically decreases performance slightly, suggesting that the neural network also includes other parts of the ECG to stabilize the model to ensure that it is analyzing the correct part of the ECG.

#### Ethics

We confirm that all experiments were performed in accordance with Helsinki guidelines and regulations of the Danish Regional Committees for Medical and Health Research Ethics. The data studies were approved by the ethical committee of Region Zealand (SJ-113, SJ-114, SJ-191), ethical committee of Copenhagen Amt (KA 98 155). Written informed consent was obtained from all study participants.

## Supplementary Information

Supplementary Information.

## Data Availability

The data is not available to the public.

## Code availability

The code used to conduct the experiments and generated the related attention maps is available on GitHub at https://github.com/stevenah/ecg-attention-maps.
