# Supplementary material for: Explaining deep neural networks for knowledge discovery in electrocardiogram analysis
Source: Sci Rep. 2021 May 26;11:10949. doi: 10.1038/s41598-021-90285-5 (PMC8154909; doi:10.1038/s41598-021-90285-5)
Supplement: Supplementary file 1 — Supplementary Information. [file 41598_2021_90285_MOESM1_ESM.docx]

# Explaining Deep Neural Networks for Knowledge Discovery in Electrocardiogram Analysis

# Steven A. Hicks^1,2,+,*^, Jonas L. Isaksen^3,+^, Vajira Thambawita^1,2^, Jonas Ghouse^3^, Gustav Ahlberg^3^, Allan Linneberg^3^, Niels Grarup^3,4^, Inga Strümke^1^, Christina Ellervik^3^, Morten Salling Olesen^3^, Torben Hansen^3,4^, Claus Graff^5^, Niels-Henrik Holstein-Rathlou^3^, Pål Halvorsen^1,2^, Mary M. Maleckar^6^, Michael A. Riegler^1,+^, and Jørgen K. Kanters^3,+^

^1^SimulaMet, 0167 Oslo, Norway

^2^Oslo Metropolitan University, 0167 Oslo, Norway

^3^University of Copenhagen, 2200 Copenhagen N, Denmark

^4^Novo Nordisk Foundation Center for Basic Metabolic Research, 2200 Copenhagen N, Denmark

^5^Aalborg University, 9220 Aalborg Ø, Denmark

^6^Simula Research Laboratory, 1364 Fornebu, Norway

*Correspondence to: steven@simula.no

^+^these authors contributed equally to this work

**Supplementary Figure S1.** Attention maps from intermediate convolutions. First row is last convolution in the first residual block, second row is last convolution in second residual block etc.

|  | T-wave morphologies MCS (rmae) | | | | QRS ≥ 120 | PR > 220 |
| --- | --- | --- | --- | --- | --- | --- |
|  | Q1 | Q2 | Q3 | Q4 | rmae | rmae |
| QT | 3.10±0.43 | 2.93±0.20 | 3.00±0.16 | 3.49±0.13 | 4.08±0.21 | 3.17±0.26 |
| PR | 2.82±0.07 | 2.70±0.06 | 2.57±0.06 | 2.83±0.08 | 3.92±0.31 | 5.58±0.70 |
| QRS | 2.49±0.07 | 2.59±0.08 | 2.58±0.10 | 2.64±0.09 | 6.04±0.25 | 4.38±0.85 |
| HR | 1.24±0.08 | 1.47±0.07 | 1.64±0.07 | 1.95±0.08 | 1.66±0.06 | 2.54±0.17 |
| STJ | 5.57±0.15 | 5.69±0.12 | 5.69±0.15 | 6.12±0.13 | 11.57±0.42 | 6.24±0.35 |
| TPEAK | 5.74±1.48 | 5.69±1.33 | 5.22±1.22 | 6.55±1.25 | 14.01±1.44 | 5.60±1.50 |
| RPEAK | 8.04±1.20 | 8.37±1.12 | 7.92±1.20 | 9.07±0.98 | 19.87±3.43 | 8.77±1.21 |

**Supplementary Table S1.** Subgroup analyses of deep learning models for electrocardiogram median beat measurement. Numbers reported are the mean absolute error and standard deviation across the five folds evaluated on the validation set (Inter99). See figure 1 for a description of the ECG markers.
